# Edaravone Dexborneol Downregulates Neutrophil Extracellular Trap Expression and Ameliorates Blood-Brain Barrier Permeability in Acute Ischemic Stroke

**DOI:** 10.1155/2022/3855698

**Published:** 2022-08-18

**Authors:** Yuanxiang Huang, Xiangjian Zhang, Cong Zhang, Wenting Xu, Wenshuo Li, Zixin Feng, Xianglan Zhang, Keke Zhao

**Affiliations:** ^1^Department of Neurology, Second Hospital of Hebei Medical University, Shijiazhuang 050000, China; ^2^Hebei Key Laboratory of Vascular Homeostasis and Hebei Collaborative Innovation Center for Cardio-Cerebrovascular Disease, Shijiazhuang 050000, China

## Abstract

**Background:**

Our previous work has shown that inflammatory processes play a detrimental role in the pathophysiology of acute ischemic stroke (AIS). Neutrophil extracellular traps (NETs) have been recognized as a key contributor to the proinflammatory response in AIS and could aggravate blood-brain barrier (BBB) damage. Recently, experimental and clinical researches showed that Edaravone Dexborneol (Eda.B), which is comprised of two active ingredients, Edaravone and (+)-Borneol, was effective in treatment of AIS. However, it is not clear whether the effects of Eda.B against AIS are related to NETs and BBB permeability.

**Methods:**

Experiment 1 was to detect the effects of Eda.B in AIS patients. Serum samples of volunteers and AIS patients were collected before and 3 days after Edaravone Dexborneol treatment. Markers of NETs and occludin were detected by ELISA kit. Experiment 2 was to explore the effects of Eda.B on experimental stroke mice. Male C57BL/6 mice were subjected to distal middle cerebral artery occlusion (MCAO) and treated with vehicle, Eda.B, or DeoxyribonueleaseI (DNase I). After stroke, the neurobehavioral tests, infarct volume, and cerebral blood flow evaluation were determined. Leakage of Evans blue was to assess the integrity of BBB. Western blot, real-time quantitative polymerase chain reaction (RT-qPCR), and immunofluorescence were used to examine the expression of NETs and tight junction- (TJ-) associated proteins.

**Results:**

Eda.B significantly improved neurological function and cerebral blood flow but reduced infarct volume after experimental stroke. Eda.B downregulated level of NETs in serum samples of AIS patients and tissue samples of MCAO mouse cortex. Eda.B and DNase I alleviated BBB permeability by upregulating TJ-associated proteins.

**Conclusion:**

NETs are related to the early stage of AIS. Eda.B exerted neuroprotective effects and ameliorated BBB permeability after AIS.

## 1. Introduction

Acute ischemic stroke (AIS) is one of the most disabling and fatal diseases worldwide [1]. The blood-brain barrier (BBB) has been considered as a critical component of the homeostasis of brain microenvironment [2]. Previous study showed that reactive oxygen species (ROS) contributed to BBB breakdown and further inflammation in the brain [3]. Nuclear and granular contents released from neutrophil activation compose neutrophil extracellular traps (NETs) [4]. The nonbactericidal effect of NETs has been gradually discovered in recent years. After AIS, markers of NETs in patients' thrombi and serum usually increase [5, 6]. NETs can be cleared by DeoxyribonueleaseI (DNase I). A previous study indicated that NETs release exacerbated the breakage of BBB, while deletion of NETs could mitigate this damage [7].

As a free radical scavenger, Edaravone (molecular formula: C10H10N2O) was initially used in the clinical treatment of AIS in China and Japan [8, 9]. Later, it was approved by the U.S. Food and Drug Administration for the treatment of amyotrophic lateral sclerosis in 2017 [10]. A previous study indicated that Edaravone could scavenge active oxygens from neutrophils effectively [11]. (+)-Borneol (molecular formula: C10H18O) is a bicyclic monoterpenoid extracted from volatile oils of various herbs. It has been demonstrated to play an anti-inflammatory role in cerebral ischemia-reperfusion injury [12]. Edaravone Dexborneol (Eda.B) is the combination of Edaravone (30 mg) and (+)-Borneol (7.5 mg). This medicine was approved by the China National Medical Products Administration in July 2020 and clinically used for AIS patients in China.

A phase III clinical trial demonstrated that compared with Edaravone, Eda.B treatment within 48 h after stroke onset significantly improved functional prognosis 90 days after stroke [13]. In animal models, a previous study showed the synergetic effect of Eda.B could reduce levels of inflammatory factors and exhibit significant long-term effects after transient cerebral ischemia injury [14]. Another study indicated Eda.B could alleviate colitis and affected the expression of TJ-associated proteins [15]. Eda.B could also decrease MPO activity and the counts of leucocytes or polymorphonuclear neutrophils after acute lung injury in mice [16]. However, the effect of Eda.B on NETs and NET-induced BBB breakdown remains unclear. Our study is aimed at exploring the effects of Eda.B on NET release, BBB disruption, and transformation of TJ-associated proteins.

## 2. Method and Materials

### 2.1. Patients

The clinical part of the study was a prospective cohort involving patients admitted to the Second Hospital of Hebei Medical University from May 2021 to February 2022 due to AIS. The study had the approval of the Research Ethics Committee of the Second Hospital of Hebei Medical University (Approval Letter No. 2021-R260). The study was registered with http://www.chictr.org.cn/ (Registration number: ChiCTR2100045950).

Patients who met the following criteria were included in the study: (1) aged 25 to 85 years old, (2) clinically diagnosed with AIS, and (3) having a National Institutes of Health Stroke Scale (NIHSS) score between 3 and 20 (detailed inclusion and exclusion criteria were in the supplement materials). Patients were assigned to different groups based on their treatment. The patients who received Eda.B and conventional treatment were assigned to the Eda.B group, while patients only received conventional treatment were assigned to the conventional treatment group. The conventional treatment of stroke was according to accepted stroke guidelines. The Eda.B treatment was Edaravone Dexborneol (National Medical Products Administration approval number: H20200007; Simcere Pharmaceutical Group Co. Ltd., Nanjing, China) in sodium chloride for injection (Eda.B 37.5 mg and saline solution 0.9 g/100 ml), bid, slowly infused for 30 min. The control group was volunteers recruited from hospital who were not diagnosed with stroke and had no history of vascular disease. Informed consent of patients and controls was obtained.

### 2.2. Serum Sample and ELISA Assay

Venous blood was obtained from patients at admission within the first 48 h after stroke onset and 3 days after treatment. Blood was collected in serum tube and was precipitated for 30 minutes. Serum was prepared by centrifugation of the blood (3000 × g) for 10 min and stored at –80°C until analysis. MPO-DNA complex and citrullinated H3 (citH3) are markers of NETs. MPO-DNA complex (cell death detection ELISA kit, Roche), citH3 (Cayman Chemical, USA), and serum occludin (USCN, China) were tested by capture ELISA based on previous studies and manufacturer's instructions [17, 18].

### 2.3. Animals and Models of Stroke

Male C57BL/6 mice (weight 20–25 g, age 8–12 weeks) were purchased from Vital River (Beijing Vital River Laboratory Animal Technology, China). Animals were housed in a 12/12 h day/night cycle, had adequate food and water, and adjusted to the new environment for at least three days before surgery. The Animal Welfare and Ethics was approved by the Research Ethics Committee of the Second Hospital of Hebei Medical University.

Focal cerebral cortical ischemia was conducted by permanent blockage of the middle cerebral artery (MCA) and the common carotid artery (CCA). The specific steps of the distal middle cerebral artery occlusion (dMCAO) have been described previously [19]. Briefly, mice were anesthetized by intraperitoneal injection of avertin (400 mg/kg, Sigma-Aldrich, USA). The right common carotid artery was isolated and permanently ligated with a surgical suture. Then, a small hole was drilled in the skull with a high-speed dental drill to expose the right distal MCA. Finally, the distal MCA was coagulated with a cauterizer (Bovie, USA) without damaging the brain surface. Mice in the Sham group had the CCA isolated but not occluded, following the small hole to expose distal MCA but not coagulated.

### 2.4. Experimental Groups and Drug Administration

Animals were randomly assigned to four groups: Sham-operated and vehicle-treated group (Sham group), distal middle cerebral artery occlusion+vehicle-treated group (MCAO group), distal middle cerebral artery occlusion+Edaravone Dexborneol-treated group (Eda.B group), and distal middle cerebral artery occlusion+DNase I-treated group (DNase I group).

Edaravone Dexborneol was provided by Simcere Pharmaceutical Group Co. Ltd. Mice in the Eda.B group received intravenous (i.v.) Eda.B at 7.5 mg/kg instantly and every 12 h after MCAO. The dose of medicine was based on a previous study [14]. Mice in the DNase I group were injected intravenous (i.v.) Deoxyribonuclease I (Solarbio, China) at 10 mg/kg instantly and every 12 h after MCAO. Mice in the Sham group and MCAO group were injected with the same amount of normal saline at the same time. At days 1 and 3 after MCAO, according to the experimental plan, animals were killed quickly by decapitation under deep anesthesia, and samples were collected for follow-up studies.

### 2.5. Neurobehavioral Tests

The rotarod test and modified neurological severity score (mNSS) were used to assess neurological function after experimental stroke as previously described [19]. The rotarod test was to evaluate physical coordination and motor learning. Before surgery, mice in all groups were continuously practiced for 3 days. For the test, mice were placed on the rod with an acceleration function (speed from 4 to 40 rpm in 4 min). The final score was presented as the average drop time of three times. The mNSS is a comprehensive scoring system that mixes sensory, motor, reflexes, and balance. The scores are ranging from 0 (normal score) to 18 (maximal deficit score). Neurobehavioral tests were performed by researchers blinded to the experimental design.

### 2.6. Infarct Volume

The infarct volume was presented by staining with 2% 2,3,5-triphenyltetrazolium chloride (TTC) 72 h after MCAO. The mouse brain was isolated and sliced into 2 mm thick coronal slices. After 30 min of 2% TTC staining at 37°C, the brain slices were fixed in 4% paraformaldehyde (PFA) overnight. Normal areas were stained red, while the infarct areas remained pale. The infarct size was calculated by the ImageJ software. Percentage hemisphere lesion volume = {[total infarct volume − (volume of intact ipsilateral hemisphere − volume of intact contralateral hemisphere)]/contralateral hemisphere volume} × 100%.

### 2.7. Evans Blue Extravasation

Evans blue (2% in saline, 4 ml/kg; Sigma, USA) was intravenously injected 2 hours before the mice were anesthetized. The brain was then extracted, and some representative images were taken. Then, the brain tissue of the ipsilateral and contralateral hemisphere was homogenized in 1 ml trichloroacetic acid and was centrifuged (21000 × g) for 20 min. Quantitative analysis of the concentration of Evans blue was estimated by evaluating the absorbance in supernatant at 610 nm.

### 2.8. Determination of ROS Content

The tissue homogenate was collected from the mouse brain. Then, assay kits (Jiancheng Bioengineering Institute, China) were used to measure glutathione peroxidase (GSH-Px) and superoxide dismutase (SOD) contents according to the manufacturer's protocol. In brief, samples were processed with water soluble tetrazole (WST) work solution and enzyme work solution from the kit. Then, the absorbance was measured at 450 nm using a microplate reader.

### 2.9. Cerebral Blood Flow Evaluation

The cerebral blood flow (CBF) was evaluated with the laser speckle contrast imaging system (PeriCam System; PERIMED, Stockholm, Sweden) based on previous research [20]. Briefly, the skin was severed to expose the skull before test. The probe was sent over the detected frontoparietal cortex area. The flows before and 5 min, 1 day, 3 days after MCAO were recorded, respectively. Color-coded images represented different levels of perfusion, and the areas of CBF in the images were calculated with the software PIMSoft (PeriCam System; PERIMED, Stockholm, Sweden).

### 2.10. Real-Time Quantitative Polymerase Chain Reaction (RT-qPCR)

Total RNA from the mouse cortex was extracted with the Animal Total RNA Rapid Extraction Kit (Generay Biotech, China), and cDNA was synthesized with All-in-One First-Strand cDNA Synthesis kit (GeneCopoeia, China). Amplification was performed with All-in-One qPCR Mix kit (GeneCopoeia, China) in LightCycler 480 (Roche Life Science, Switzerland). Primer sequences are presented in supplementary table 1.

### 2.11. Western Blot

Mouse cortical proteins were extracted using Radio Immunoprecipitation Assay (RIPA) lysis buffer (Solarbio, China). The protein concentration was quantified by a bicinchoninic acid protein assay reagent kit (Thermo Fisher Scientific, USA). Then, equivalent amounts of protein (50 *μ*g) with buffer were loaded in gels and separated by SDS-PAGE. Next, proteins were transferred onto polyvinylidene fluoride (PVDF) membrane (Roche, USA) and blocked with 5% nonfat milk for 1 h at room temperature, followed by incubation with primary antibodies specific for occludin (Invitrogen, US), claudin5 (Bioworld Technology, USA), TNF*α* (Santa Cruz, USA), Histone H3 (Abcam, UK), citH3 (citrulline R2+R8+R17) (Abcam, UK), and *β*-actin (Cell Signaling Technology, USA) overnight at 4°C. After being washed three times with TBST, the membrane was incubated with anti-rabbit IgG secondary antibody (Rockland, USA) or anti-mouse IgG secondary antibody (Rockland, USA) for 1 h at room temperature and then was washed with TBST for another three times. Finally, the membrane was scanned by an infrared scanner (LICOR Bioscience, USA). The intensity of the bands was calculated by the ImageJ software.

### 2.12. Immunofluorescence Staining

Mice were anesthetized with avertin and were perfused transcardially with saline quickly to clear blood, followed by 4% PFA in 0.1 M phosphate-buffered saline (PBS). Then, the brain was extracted and was dehydrated in 30% sucrose for 48 h. Frozen coronal brain slices were sectioned 20 *μ*m thick by using a cryotome (Thermo Scientific, USA). The sections were permeabilized with 0.3% Triton X-100 for 15 min and were blocked with 10% normal donkey serum for 1 h at 37°C, followed by incubated with primary antibodies occludin (Invitrogen, US), claudin5 (Bioworld Technology, USA), CD31 (BDBiosciences, USA), citH3 (citrulline R2+R8+R17) (Abcam, UK), and Ly6G (BDBiosciences, USA) overnight at 4°C. Next, slices were washed three times with PBS and incubated with appropriate secondary antibodies (Alexa Fluor 488 or 594, Jackson Immuno Research, USA) at 37°C for 1 h. Finally, slices were washed with PBS for another three times and stained with DAPI. Images were taken with laser scanning confocal microscopy (Zeiss LSM880, German).

### 2.13. Statistical Analyses

All statistical analyses were performed by the SPSS 26 software (IBM, USA). Continuous data were presented as the mean ± standard deviation (SD). All of the data were tested for normality and variance homogeneity. Normally distributed data were analyzed using a one-way analysis of variance (ANOVA), while nonnormally distributed data were analyzed with Mann–Whitney *U* or Kruskal–Wallis test. The post hoc analysis in ANOVA was LSD test (equal variances assumed) or Tamhance's T2 test (equal variances not assumed). Multiple linear regression analysis was performed to evaluate the independent risk factors for serum occludin in patients with acute ischemic stroke. *P* < 0.05 was defined as the statistically significant level.

## 3. Result

### 3.1. Edaravone Dexborneol Reduced the Levels of Serum NETs and Occludin in Patients after Stroke

**Figure 1:**
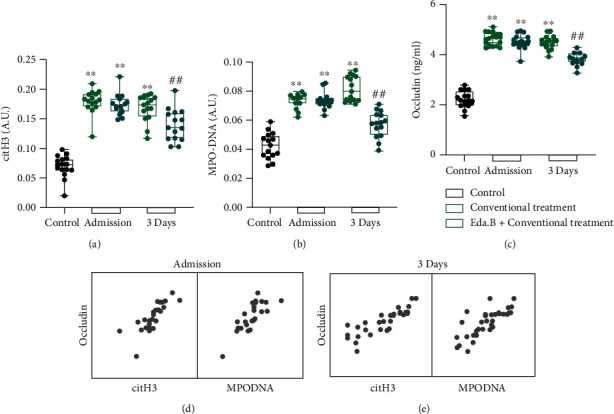
Levels of NET markers and occludin level in patients' serum sample. (a–c) citH3 and MPO-DNA complex are expressed in arbitrary units (A.U.), and the occludin concentration is expressed as ng/ml. (d, e) Matrix scatter plot of correlation between citH3, MPO-DNA complex, and occludin in stroke patients. ^∗∗^*P* < 0.01 (conventional treatment group or Eda.B group at admission vs. control group) and ^##^*P* < 0.01 (Eda.B group vs. conventional treatment), *n* = 15 in each group.

Details of the patients' characteristics are described in supplementary table 2. The results of ELISA test showed that compared with the control group, serum levels of occludin, MPO-DNA, and citH3 in the conventional treatment group and Eda.B group were higher after stroke onset (*P* < 0.01), and there was no significant difference between the two groups (*P* > 0.05). Contrasted with the conventional treatment group, the serum levels of occludin, MPO-DNA, and citH3 in the Eda.B group decreased on the third day after being hospitalized (*P* < 0.01) (Figures 1(a)–1(c)). To explore whether citH3 or MPO-DNA complex affected occludin level in AIS patients, multiple linear regression analyses were performed. It was revealed that the citH3 and MPO-DNA accounted for 67.4% of the variation of serum occludin level in patients at admission and 75.4% in patients 3 days after treatment. CeitH3 and MPO-DNA which were correlated with serum occludin were included as independent variables, and occludin level was served as dependent variables (Figures 1(d) and 1(e) and Tables 1 and 2). These results suggested that Eda.B could reduce the serum levels of NETs and occludin induced by AIS. Serum occludin level was positively correlated with citH3 and MPO-DNA complex.

### 3.2. Edaravone Dexborneol Improved Neurological Function and Reduced Infarct Volume in MCAO Mice

**Figure 2:**
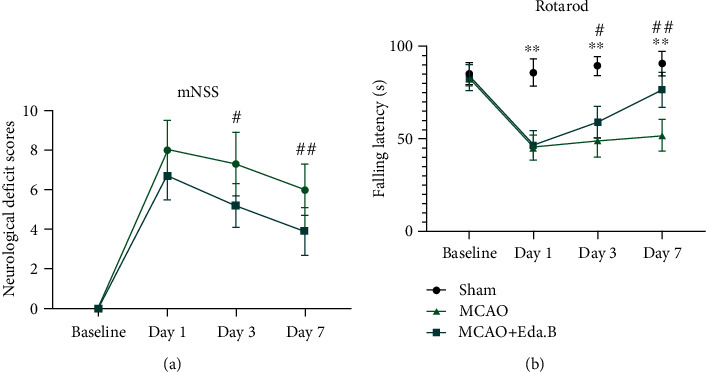
Eda.B improved neurological recovery in ischemic mice. (a) The mNSS evaluation at baseline and days 1, 3, and 7 after MCAO. (b) Rotarod test evaluation in Sham group, MCAO group, and Eda.B group at baseline and days 1, 3, and 7 after MCAO. ^∗∗^*P* < 0.01 (MCAO group vs. Sham group), ^#^*P* < 0.05 (Eda.B group vs. MCAO group), and ^##^*P* < 0.01 (Eda.B group vs. MCAO group), *n* = 10 in each group.

To further verify whether Eda.B could improve brain damage after stroke, rotarod test and mNSS were conducted to evaluate neurological function before and at days 1, 3, and 7 after MCAO. At day 1, there was no significant difference among all the experimental groups (*P* > 0.05). Compared with mice in the MCAO group, mice received Eda.B showed significantly higher scores in the rotarod test and less mNSS at day 3 (*P* < 0.05) and day 7 (*P* < 0.01) after MCAO (*n* = 10 in each group, Figures 2(a) and 2(b)).

**Figure 3:**
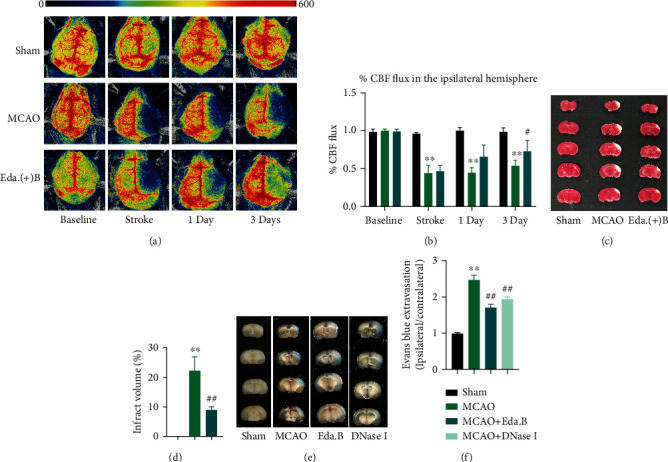
Protective effects of Eda.B on MCAO mice in cerebral blood flow (CBF), TTC-stained, and Evans blue leakage. (a) Representative perfusion images of CBF at baseline, 5 min (stroke), day 1, and day 3 after MCAO. (b) The percent CBF flux (%) in the ipsilateral hemisphere was shown at different time points. (c, d) Representative TTC-stained brain sections and quantitatively infarct volume in Sham group, MCAO group, and Eda.B group at day 3 after stroke. (e, f) Representative images and quantification data of Evans blue leakage in the brain in Sham group, MCAO group, Eda.B group, and DNase I group at day 3 after stroke. ^∗∗^*P* < 0.01 (MCAO group vs. Sham group), ^#^*P* < 0.05 (Eda.B group vs. MCAO group), and ^##^*P* < 0.01 (Eda.B group or DNase I group vs. MCAO group), *n* = 4 in each group.

The infarct volume of mouse cortex at day 3 after experimental stroke is shown in Figure 3(c). In the MCAO group, extensive cortical infarction can be found. Compared with mice in the MCAO group, those treated with Eda.B exhibited reduced infarct volume (*P* < 0.01, *n* = 5, Figures 3(c) and 3(d)). This is consistent with the results of neurological deficit scores.

### 3.3. Edaravone Dexborneol Improved CBF in the Ischemic Hemisphere

CBF in mouse cerebral cortex was analyzed by a laser speckle contrast imaging system. The typical images of each group at different time points are presented in Figure 3(a), and the quantified results are presented in Figure 3(b). Blood perfusion in the infarcted cortex was significantly reduced at all stages after MCAO. At day 3 after stroke, the CBF in ipsilateral hemisphere was more abundant in the Eda.B group compared with the MCAO group (*P* < 0.05, *n* = 4).

### 3.4. Edaravone Dexborneol Reduced the Levels of NETs and Inflammatory Factors in MCAO Mouse Cortex

**Figure 4:**
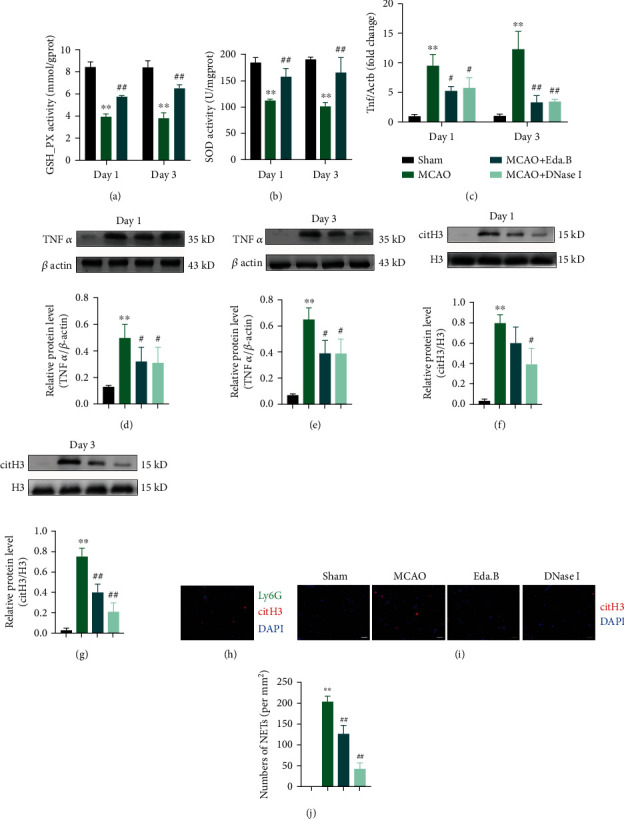
Protective effects of Eda.B against NETs and inflammatory factors. (a, b) The levels of GSH-Px and SOD in different groups were quantified by commercial kits. (c) mRNA expression of Tnf in mouse cortex at day 1 and day 3 after stroke.(d–g) Protein levels of TNF*α* and citH3 at day 1 and day 3 after stroke. Representative bands are on the top. (h) Representative images of sections stained with citH3 (red), neutrophil marker Ly6G (green), and DAPI (blue) in infarct cortex. Scale bar = 20 *μ*m. (i, j) Representative images of sections stained with citH3 (red) and DAPI (blue) in infarct cortex and quantification of NETs in different groups. Scale bar = 40 *μ*m. ^∗∗^*P* < 0.01 (MCAO group vs. Sham group), ^#^*P* < 0.05 (Eda.B group or DNase I group vs. MCAO group), and ^##^*P* < 0.01 (Eda.B group or DNase I group vs. MCAO group), *n* = 5 in each group.

The antioxidant stress effect of Eda.B was verified by measuring GSH-Px and SOD activity. Data in Figures 4(a) and 4(b) indicated that MCAO could significantly decrease the activity of GSH-Px and SOD (*P* < 0.01). However, Eda.B treatment effectively increased GSH-Px and SOD activity (*P* < 0.01).

Western blot analysis and immunostaining were adopted to test whether NETs were produced in the ischemic brain and could be affected by Eda.B. WB analysis showed an increased amount of citH3 at day 1 and day 3 after stroke in the MCAO group compared with the Sham group. DNase I could significantly reduce citH3 expressions after experimental stroke. Eda.B treatment reduced the level of citH3 at day 3 after MCAO (*P* < 0.01) (Figures 4(f) and 4(g)). And inflammatory factor tumor necrosis factor-alpha (TNF*α*) showed a similar tendency (Figures 4(c)–4(e)). Immunostaining revealed that the infarct cortex was extensively labeled with citH3-positive cells and Ly6G-positive neutrophils at day 3 after stroke (Figure 4(h)). There were no NETs detected in the Sham group while MCAO induced the production of NETs. Treatment with Eda.B and DNase I significantly reduced NET levels in mouse cortex slices (*P* < 0.01) (Figures 4(i) and 4(j)).

### 3.5. Edaravone Dexborneol Ameliorated the Blood-Brain Barrier Permeability in MCAO Mouse Cortex

To explore the effects of Eda.B on BBB permeability, extravasation of Evans blue was assessed, and the blue area illustrated BBB destruction (Figures 3(e) and 3(f)). BBB breakdown was discovered in all the MCAO, Eda.B, and DNase I groups 72 h after MCAO. However, Evans blue leakage was significantly reduced in the Eda.B group and DNase I group compared with the MCAO group (*P* < 0.01).

**Figure 5:**
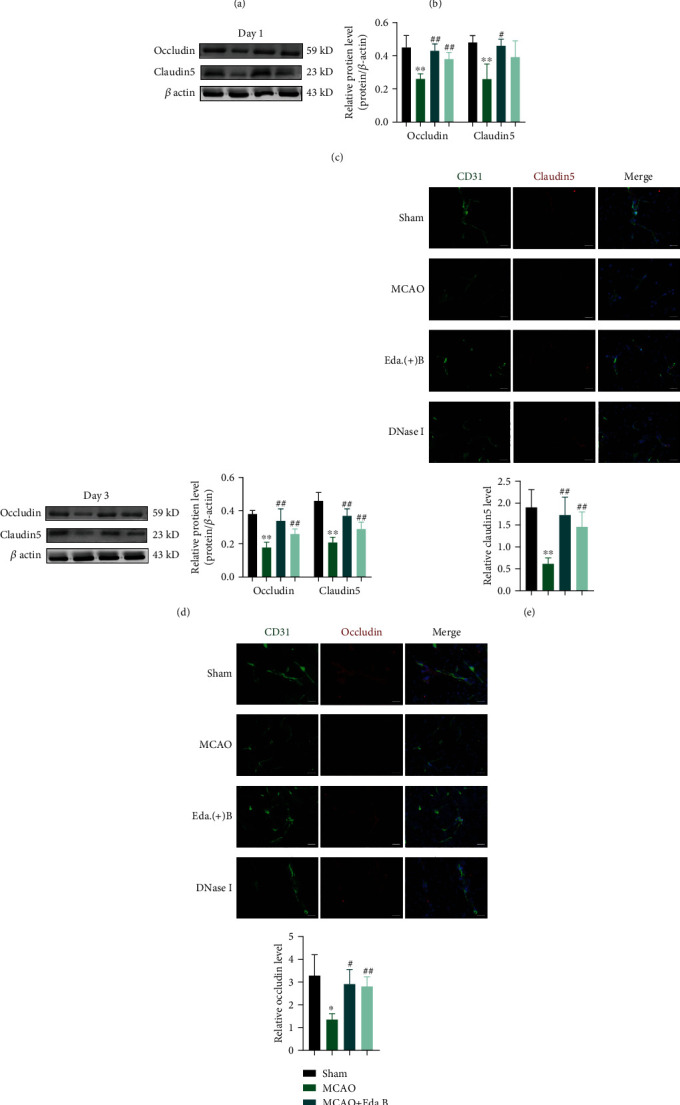
Eda.B and DNase I administration alleviated degradation of TJ-associated protein in MCAO mouse cortex. (a, b) mRNA expression of occludin and claudin5 in mouse cortex at day 1 and day 3 after stroke. (c, d) Protein levels of occludin and claudin5 at day 1 and day 3 after stroke. Representative bands are on the left. (e, f) Representative images of sections stained with claudin5 or occludin (red), CD-31 (green), and DAPI (blue). Scale bar = 20 *μ*m. Mean fluorescence intensity is on the bottom. ^∗^*P* < 0.05 (MCAO group vs. Sham group), ^∗∗^*P* < 0.01 (MCAO group vs. Sham group), ^#^*P* < 0.05 (Eda.B group or DNase I group vs. MCAO group), and ^##^*P* < 0.01 (Eda.B group or DNase I group vs. MCAO group), *n* = 5 in each group.

Next, we examined TJ-associated genes and proteins by RT-qPCR and WB. The mRNA expressions of occludin and claudin5 were significantly downregulated at day 1 and day 3 after MCAO compared with the Sham group (Figures 5(a) and 5(b)). Eda.B or DNase I treatment significantly increased the mRNA expressions of claudin5 at day 1 and day 3 after MCAO. Almost consistently, Western blot results indicated that the protein expressions of occludin and claudin5 were reduced at day 1 and day 3 in the MCAO group. Eda.B or DNase I treatment ameliorated MCAO-induced changes of occludin and claudin5, except for claudin5 in the DNase I group at day 1 (Figures 5(c) and 5(d)).

Confocal microscopy revealed that claudin5 and occludin were colocalized with CD31, which is used to label the microvessels, in mouse brain slices. The mean fluorescence intensity of claudin5 and occludin was decreased significantly at day 1 and day 3 after stroke. Eda.B and DNase I could attenuate the decrease of claudin5 and occludin protein levels after MCAO (Figures 5(e) and 5(f)). Together, these results showed that Eda.B and degradation of NETs with DNase I could reduce BBB permeability after stroke in mice.

## 4. Discussion

Previous studies about Eda.B could be divided into two stages. Before 2020 when Eda.B was widely used clinically, researchers first identified the optimal ratio of Edaravone and (+)-Borneol (4 : 1) and later explored its anti-inflammatory effects on colitis, acute lung injury, and stroke [15, 16]. For stroke, it was revealed that Eda.B could scavenge free radicals, decrease infarct area, and inhibit proinflammatory mediator expression in rats [14]. After 2020, more researches about Eda.B on stroke and subarachnoid hemorrhage were conducted [21, 22]. These studies still focused on the anti-inflammatory effects of Eda.B. Our study further documented a range of protective effects of Eda.B via samples from MCAO mice and stroke patients. We demonstrated for the first time that (1) Eda.B reduces the levels of NETs after stroke in patients' serum sample and mouse cortex. (2) Eda.B alleviates the BBB permeability and improves blood flow after stroke.

Inflammation and oxidative stress are vital contributors to the pathological process of brain injury after stroke, and anti-inflammation therapy plays an important role in the early phase of AIS [23]. NETs release many cytotoxic proteases and induce endothelial cell damage and disrupt vascular homeostasis [24]. Consistently, our results showed that stroke activated excessive release of NETs. Inhibition of NETs with DNase I or Eda.B injection significantly reduced the extent of inflammation and BBB damage. These findings suggested that Eda.B may be beneficial to NET-dependent vascular destabilization during the early stage after stroke. Level of NETs is elevated after stroke. However, the variation tendency of NETs content in infarcted cortex remains controversial. Kang et al.'s study showed an increased amount of citH3 mostly robust from 3–5 days and reached its peak at day 3, suggesting that NETs may come into play during the delayed stage after stroke [7], while another study showed that NETs reached its highest point at day 1 after stroke [25]. Our research did not find a prominent difference in citH3 content between day 1 and day 3 after stroke.

The generation of NETs in neutrophils is complex. In short, it can be divided into three types: suicidal NETs, vital NETs, and mitochondrial NETs [26]. Suicidal NETs are dependent on NADPH-dependent ROS and ultimately lead to neutrophil death [27]. This process can be blocked by certain NADPH oxidase inhibition or ROS scavengers [28]. As a ROS scavenger, the effect of Eda.B on NETs may be mainly reflected in suicidal NETs. Vital NETs occur without neutrophil death, and mitochondrial NETs are composed when mitochondrial DNA was released. Peptidylarginine deiminase 4 (PAD4) is also essential for the NET formation via histone citrullination. In PAD4-/- mice or mice injected Cl-amidine (PAD4 inhibitor), levels of NETs significantly decreased [29].

TJ-associated proteins are important structural components of BBB to maintain its integrity. After ischemic stroke, fragments of cleaved occludin could release into circulation. The serum concentration of occludin correlates with the extent of BBB disruption, which means serum occludin level could be regarded as a potential biomarker of AIS [30]. Specific intervention, such as normobaric hyperoxia, could reduce the blood occludin level after stroke [31]. Our result showed that serum occludin increased in AIS patients, and stroke decreased the level of TJ-associated proteins in perivascular space of mouse cortex. Eda.B or DNase I treatment reversed this tendency and alleviated the BBB permeability. NETs could affect the level of TJ-associated proteins after stroke. ZO-1, claudin5, and occludin levels were increased in PAD4-/- mice and mice given Cl-amidine [7]. Our multiple linear regression analyses indicated a strong correlation between NETs and serum occludin levels in patients, which is consistent with results in mice. However, more research on this topic needs to be undertaken before the association between NETs and TJ-associated proteins is more clearly understood.

One limitation is that the signaling pathways involved in the effects of Eda.B on stroke were not explored. As a new synthetic drug, its specific pathways are not clear enough. A previous study demonstrated that Eda.B could promote M2 macrophage polarization via the JAK2-STAT3 signaling pathway [15]. A recently published study pointed that Eda.B could stimulate the Nrf2/HO-1 pathway to inhibit NADPH oxidase 2 expression in mice after stroke [22]. Considering the relative mechanism of NET production, the apoptotic pathways may play an important role in the pharmacological effect of Eda.B. Transcriptomics and proteomics are needed for further research.

## 5. Conclusion

NETs exacerbated BBB damage in the early stage of acute ischemic stroke. In stroke patients, Eda.B reduced the serum levels of NETs and occludin, and occludin level was positively correlated with NETs. In animal models, Eda.B exerted protective effects on neurological function, cerebral blood flow, and could reduce the levels of NETs and other inflammatory factors, attenuating BBB permeability. The protective effects of Eda.B are possibly related to its ability to reduce NETs level.

## Figures and Tables

**Table 1:** Multiple linear regression analysis of citH3 and MPO-DNA affecting serum occludin levels in AIS patients at admission.

	Unstandardized coefficients		Standardized coefficients		
	*B*	SE	Beta	*t*	*P* value
citH3	6.800	2.430	0.447	2.798	<0.01
MPO-DNA	1.694	0.608	0.445	2.786	0.01

The dependent variable was occludin (adjusted *R*^2^ = 0.674).

**Table 2:** Multiple linear regression analysis of citH3 and MPO-DNA affecting serum occludin levels in AIS patients at day 3 after treatment.

	Unstandardized coefficients		Standardized coefficients		
	*B*	SE	Beta	*t*	*P* value
citH3	6.915	1.705	0.573	4.056	<0.01
MPO-DNA	8.313	3.257	0.360	2.553	0.02

The dependent variable was occludin (adjusted *R*^2^ = 0.754).

## Data Availability

The data used to support the findings of this study are included within the article.

## Supplementary Materials

Supplementary Materials Supplementary Table 1: summary of the RT-qPCR primer sequences. Supplementary Table 2: baseline characteristics of the stroke patients and volunteers.
