# Supplementary material for: Edaravone Dexborneol Downregulates Neutrophil Extracellular Trap Expression and Ameliorates Blood-Brain Barrier Permeability in Acute Ischemic Stroke
Source: Mediators Inflamm. 2022 Aug 18;2022:3855698. doi: 10.1155/2022/3855698 (PMC9410976; doi:10.1155/2022/3855698)
Supplement: Supplementary Materials — Supplementary Table 1: summary of the RT-qPCR primer sequences. Supplementary Table 2: baseline characteristics of the stroke patients and volunteers. [file 3855698.f1.docx]

**Supplement materials**

The detailed inclusion criteria were: (1) 25–85 years of age; (2) meeting the diagnostic criteria for stroke; (3) admitted to the hospital within 48 h of stroke onset; (4) new infarct lesion found by head CT scan or MRI diffusion-weighted imaging. The exclusion criteria were: (1) cerebral infarction definitely due to other causes after auxiliary examinations (such as tumors, parasitic infections, vascular malformations, and immune system diseases); (2) history of allergy to edaravone dexborneol; (3) underwent thrombectomy after admission; (4) death within 72 h after admission; (5) severe liver and renal dysfunction; (6) confirmed mental illness; (7) women during pregnancy and lactation.

| Primers | Forward (5’ - 3’) | Reverse (5’ - 3’) |
| --- | --- | --- |
| Tnf | AGCCGATGGGTTGTACCTTG | ATAGCAAATCGGCTGACGGT |
| Ocln | GTCCAATGGCCTACTCCTCC | CTTCAGGCACCAGAGGTGTT |
| Cldn5 | CTGCCTTCCTGGACCACAAC | CGCCAGCACAGATTCATACAC |
| Actb | CTAAGGCCAACCGTGAAAAG | ACCAGAGGCATACAGGGACA |

[Supplementary](javascript:;) [Table](javascript:;) 1. Summary of the RT-qPCR Primers sequences.

| Group | Control（n=15） | Conventional（n=15） | Eda.B（n=15） |
| --- | --- | --- | --- |
| Age (years) | 55.2±3.2 | 62.1±2.6 | 62.8±3.4 |
| Gender(female/male) | 7/8 | 5/10 | 4/11 |
| current smoker(n) | 5 | 6 | 6 |
| hypertension(n) | 8 | 13 | 13 |
| diabetes(n) | 5 | 4 | 4 |
| NIHSS | -- | 5.1±0.8 | 6.5±0.7 |

[Supplementary](javascript:;) [Table](javascript:;) 2. Baseline characteristics of stroke patients and volunteers.
